# Microemulsion and Sol-Gel Synthesized ZrO_2_-MgO Catalysts for the Liquid-Phase Dehydration of Xylose to Furfural

**DOI:** 10.3390/molecules22122257

**Published:** 2017-12-18

**Authors:** Almudena Parejas, Vicente Montes, Jesús Hidalgo-Carrillo, Elena Sánchez-López, Alberto Marinas, Francisco J. Urbano

**Affiliations:** Department of Organic Chemistry, Institute for Research in Fine Chemistry and Nanochemistry, IUIQFN, Universidad de Córdoba, Campus de Rabanales, Marie Curie Building, E-14014 Córdoba, Spain; q12pabaa@uco.es (A.P.); vicente.montesjimene@ucalgary.ca (V.M.); q12hicaj@uco.es (J.H.-C.); g02saloe@uco.es (E.S.-L.)

**Keywords:** xylose dehydration, furfural, MgO-ZrO_2_ mixed catalysts, microemulsion synthesis, sol-gel synthesis, solvent effect

## Abstract

Two series of catalysts were prepared by sol-gel and microemulsion synthetic procedure (SG and ME, respectively). Each series includes both pure Mg and Zr solids as well as Mg-Zr mixed solids with 25%, 50% and 75% nominal Zr content. The whole set of catalysts was characterized from thermal, structural and surface chemical points of view and subsequently applied to the liquid-phase xylose dehydration to furfural. Reactions were carried out in either a high-pressure autoclave or in an atmospheric pressure multi-reactor under a biphasic (organic/water) reaction mixture. Butan-2-ol and toluene were essayed as organic solvents. Catalysts prepared by microemulsion retained part of the surfactant used in the synthetic procedure, mainly associated with the Zr part of the solid. The MgZr-SG solid presented the highest surface acidity while the Mg3Zr-SG one exhibited the highest surface basicity among mixed systems. Xylose dehydration in the high-pressure system and with toluene/water solvent mixture led to the highest furfural yield. Moreover, the yield of furfural increases with the Zr content of the catalyst. Therefore, the catalysts constituted of pure ZrO_2_ (especially Zr-SG) are the most suitable to carry out the process under study although MgZr mixed solids could be also suitable for overall processes with additional reaction steps.

## 1. Introduction

Nowadays, one of the priorities of scientists is the search for alternatives to non-renewable energies. A renewable solution for this challenge is biomass, which can provide both energy [1] and useful chemical compounds for industry [2].

The estimated biomass production is about 10^11^ ton per year whose 60% is terrestrial and 40% aquatic. However, only 3% of the produced biomass is utilized for human-derived applications [3]. Therefore, the transformation of biomass into platform molecules that can be further converted into high-added value chemicals is a hot topic nowadays [4,5]. 

Biomass is composed by carbohydrates (75%), lignin (20%) and a mixture of triglycerides, proteins and terpenes (5%) [5]. Lignocellulose is the main constituent of plant cell walls whose hydrolysis yields a mixture of C5 and C6 sugars as well as aromatic compounds (lignin). Therefore, valorization of pentoses and hexoses, such as xylose or glucose, is very interesting from the chemical point of view [4].

Furfural is one of the top platform molecules that can be obtained from biomass [6,7] and that is able to be further transformed into a wide range of chemicals by chemo and/or enzymatic transformations. It is a natural precursor to a range of furan-based chemicals and solvents such as methyltetrahydrofuran, tetrahydrofuran, tetrahydrofurfuryl alcohol and furoic acid, among others. Compounds derived from furfural are used in the pharmaceutical industry, and as plastics, agricultural fungicides or nematocides, lubricants, resins, bleaching agents, food and beverage additives, wood modifiers or book preservatives, among other uses. Moreover, within the last decade the conversion of furfural into diesel by means of a combination of dehydration, aldol condensation and hydrogenation reactions has been described [8,9,10,11,12].

Due to the continuous demand for furfural, the search for alternative synthetic routes or the improvement of the existing ones is still an active research area. The classic industrial furfural production process is based on the treatment of pentose-containing lignocellulosic biomass with mineral acids (H_2_SO_4_, HCl) [13]. However, mineral acids cause corrosion of the reactors and generate toxic effluents; therefore, the design of active, selective and stable heterogeneous solid acid catalysts is a key point to reduce costs of reactors as well as the environmental impact of the process. An additional effort should be made to improve the process sustainability by designing water resistant and recyclable solid acid catalysts. Furthermore, if a chemical process is to be coupled following the synthesis of furfural in the so-called cascade processes, it may be necessary to synthesize catalysts which, in addition to acidic properties, have a second functionality which may be basic sites (e.g., for condensation reactions) or metal centers (for redox processes) [14].

During the process, due to the high reactivity of furfural molecule, several side reactions such as condensation or acetalyzation can take place leading to hemiacetals and oligomers (humines) [15,16,17]. Additionally, xylose fragmentation reactions can occur producing acetol, lactic acid, glyceraldehyde, etc. [18]. Water is typically the solvent of choice for sugar transformation reactions due to solubility, economic and environmental aspects. However, water favors the formation of humines thus lowering the selectivity to the desired product. An adequate solvent mixture (biphasic systems) can help to reduce these by-products since formed furfural could be rapidly extracted to a second liquid phase, thus avoiding further transformation [19,20,21]. Toluene, *n*-butanol or ethyl butyrate [22,23] can be found among the secondary solvents (extracting agents) reported in the literature. Alternative described solutions such as the use of ionic liquids [24] or supercritical carbon dioxide [25] are expensive or technologically demanding.

This piece of research deals with the synthesis and characterization of zirconium/magnesium mixed oxides in order to obtain bifunctional acid/base solids. Two series of catalysts were synthesized using the sol-gel or microemulsion processes (SG and ME series, respectively). The solids were subsequently tested for xylose dehydration to furfural. The final goal was to obtain some robust and water-resistant acid-base catalysts that can be active not only for xylose dehydration to furfural but also able to carry out one-pot or cascade reactions for further transformation of furfural [26].

## 2. Results

### 2.1. Catalysts Characterization 

**Figure 1:**
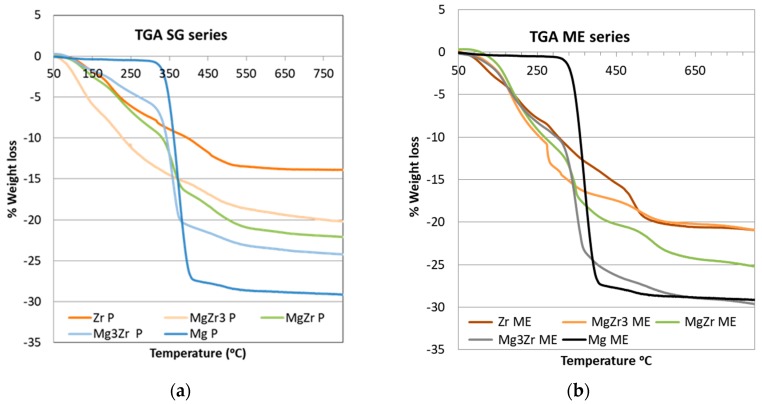
Thermogravimetric analysis (TGA) of the dried gels corresponding to both series of solids synthesized: (**a**) Sol-Gel and (**b**) Microemulsion.

Thermogravimetric analysis (TGA) was carried out for the dried gels before thermal treatment at 200 °C, the obtained results being presented in Figure 1. The TGA profiles obtained for both series of solids were quite similar. As for pure systems, the weight loss for both Mg-SG and Mg-ME systems was around 29% with a single weight loss starting at temperatures around 325 °C. This weight loss is consistent with the transformation from Mg(OH)_2_ to MgO (theoretical weight loss around 31%). For pure Zr-SG and Zr-ME solids a more complex profile was obtained with several weight loss spreading over 100–500 °C temperature range. Overall, the weight loss was 13.9% and 20.8% for Zr-SG and Zr-ME, respectively. Taking into account that the theoretical weight loss associated to the transformation of ZrO(OH)_2_ into ZrO_2_ is around 13%, the weight loss of 20.8% obtained for the Zr-ME gel has to be also ascribed to the burning of the surfactant remaining on the surface of the solid. On the other hand, the fact that the weight loss for Mg-ME and Mg-SG solids is very similar and close to the theoretical value suggests that no surfactant is remaining on the surface of pure Mg-ME solid. Finally, mixed MgZr solids in both series exhibited an intermediate behavior between both pure Zr and Mg solids.

**Figure 2:**
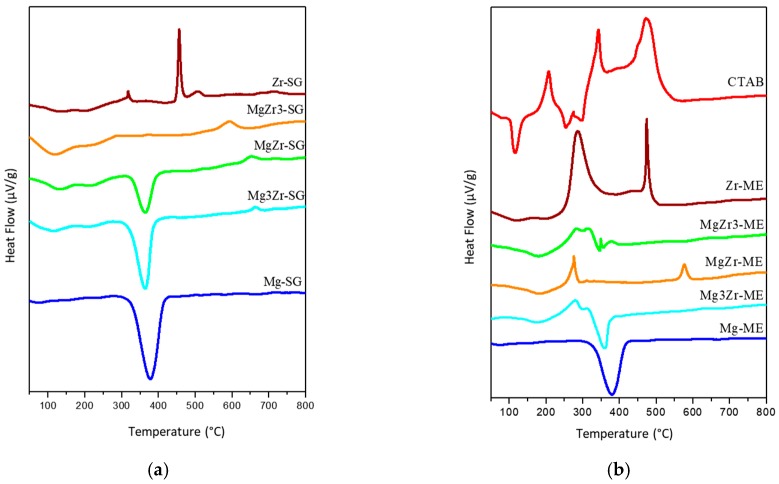
Differential thermal analysis (DSC) profiles obtained for the dried gels corresponding to both series of solids synthesized: (**a**) Sol-Gel and (**b**) Microemulsion.

Differential thermal analysis (DSC) of the solids is presented in Figure 2 where it can be observed that for pure Mg-based solids there is a single endothermic signal at 375 °C associated to the transition from Mg(OH)_2_ to MgO [27]. No differences were observed between Mg-SG and Mg-ME solids in DSC profiles, in agreement with the TGA data. On the contrary, Zr-SG and Zr-ME exhibited very different DSC profiles. The first one only presents an exothermic peak (glow exotherm) centered at 450 °C associated to the crystallization of the low-temperature tetragonal metastable phase of ZrO_2_ [28]. For mixed solids in the SG series, the glow exotherm is also observed but is weaker and shifted to higher temperatures (up to 650 °C for Mg3Zr-SG), indicating that crystallization is somehow inhibited or retarded in the presence of Mg species [29].

As far as the ME series is concerned, Zr-ME solid presents a DSC profile with two exothermic peaks; the above commented glow exotherm at 475 °C and another one at lower temperature (around 300 °C) associated to the combustion of the organic part of the surfactant remaining on the catalyst surface. This peak is smaller as the magnesium content increases, until complete disappearance for Mg-ME. Again, it seems that the amount of surfactant retained on the surface of the solids is greater for the systems containing the highest Zr content. The DSC profile for pure surfactant, CTAB, is included in Figure 2 for comparison. It is assumed that the CTAB combustion takes place at lower temperatures on the surface of the solids.

As far as the chemical composition of the solids is concerned, it was obtained from SEM-EDX and XPS measurements, the results being presented in Table 1. EDX and XPS atomic concentration values are somehow different to each other and, in this sense, it has to be taken into account that XPS penetration is lower than the EDX one. Therefore, XPS data have to be assumed as a surface composition meanwhile EDX values are associated to bulk chemical composition of the mixed solids. Thus, EDX data indicate that the magnesium content is somehow higher than the nominal value except for the MgZr3-ME solid which is the mixed solid with the lowest magnesium content. It seems that either the precipitation at pH 10 is more efficient for Mg(OH)_2_ or, more likely, precipitated ZrO(OH)_2_ species re-dissolve at the precipitating pH.

On the other hand, XPS data revealed an opposite behavior, i.e., the Zr content is higher than the nominal value for the mixed solids, except for the MgZr3-ME. This finding implies that ZrO_2_ particles are localized on the outer part of the aggregates while the MgO ones are located within the inner part of the agglomerated particles.

SEM-EDX measurements have revealed that in the solids prepared through the microemulsion technique, in spite they were washed 5 times with chloroform/ethanol, a small amount of bromine coming from surfactant molecules was detected on the solids surface (Supplementary Figure S1). In addition, as the zirconium content increases in ME series, the amount of detected Br is higher (from 0.9% for Mg-ME to 4.3% for Zr-ME), thus pointing out the higher affinity of the surfactant for the ZrO_2_ particles.

Nitrogen adsorption–desorption isotherms for the solids calcined at 200 °C were obtained (file. Red arrow marks Br signal, Supplementary Figure S2) and the BET surface area presented in Table 1. The largest surface area was obtained for pure Zr-SG solid (239 m^2^·g^−1^) while Zr-ME exhibited a somehow lower surface area (222 m^2^·g^−1^), revealing that the surfactant remaining on the catalyst negatively affects its surface area. On the other hand, pure Mg-SG and Mg-ME solids present the lowest surface area (41 and 31 m^2^·g^−1^ respectively) indicating that the porous structure is still absent on these solids after calcination at 200 °C. As far as the mixed solids are concerned, the higher the Zr content the higher the surface area. It is, however, relevant to indicate that the Mg3Zr-ME solid with a scarce 9.5% Zr (EDX, Table 1) presents a BET surface area as high as 196 m^2^·g^−1^.

**Figure 3:**
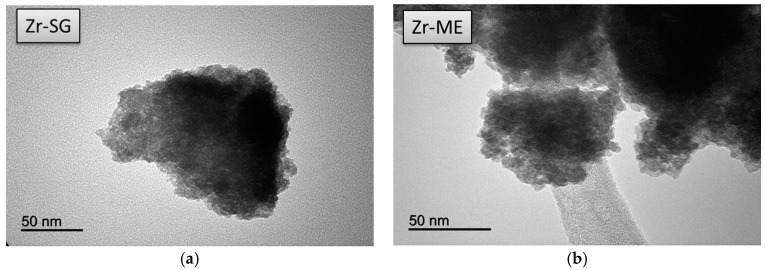
TEM images corresponding to pure (**a**) Zr-SG and (**b**) Zr-ME solids.

**Figure 4:**
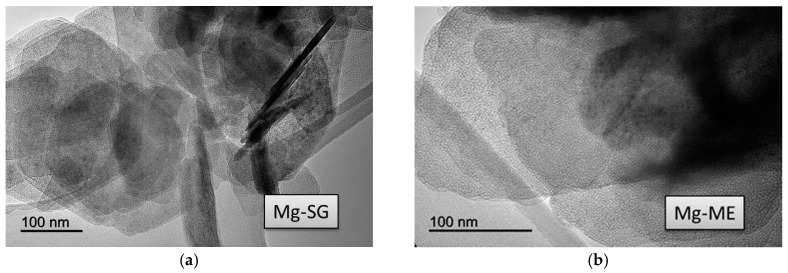
TEM images corresponding to pure (**a**) Mg-SG and (**b**) Mg-ME solids.

**Figure 5:**
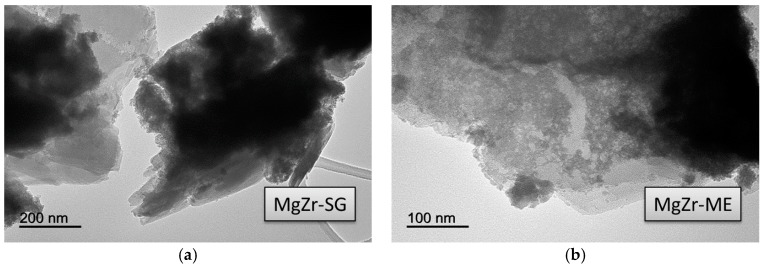
TEM images corresponding to **(a)** MgZr-SG and **(b)** MgZr-ME solids.

Transmission electron microscopy (TEM) images obtained for the solids are presented in Figure 3, Figure 4 and Figure 5. Irrespective of the synthetic procedure, pure Zr solids are constituted by agglomerated small particles (2–5 nm) (Figure 3) while pure Mg-SG and Mg-ME solids are based on larger hexagonal particles of around 200 nm in diameter (Figure 4). Moreover, mixed MgZr-SG and MgZr-ME solids are formed by large MgO particles surrounded by small ZrO_2_ nanoparticles (Figure 5), in agreement to the higher Zr content observed in the surface XPS measurements (Table 1).

### 2.2. Catalysts Surface Acid-Base Properties

Catalysts surface acidity was determined from temperature programmed desorption of pre-adsorbed pyridine (TPD-PY), the results being presented in Table 2 and Supplementary Figure S3. Values obtained for pyridine desorbed per gram of catalyst indicate that, as expected, pure Zr solids are much more acidic than pure Mg ones. Thus, Zr-SG and Zr-ME desorbed 748 and 871 µmol PY/g while Mg-SG and Mg-ME only desorbed 176 and 115 µmol/g, respectively. On the other hand, Mg-Zr mixed solids present a surface acidity in the 444–991 µmol/g range, with some interesting features that will be presented below. First of all, those mixed solids obtained from microemulsion present similar acidity, irrespective of its chemical composition—856, 885 and 788 µmol/g for Mg3Zr-ME, MgZr-ME and MgZr3-ME, respectively, while, on the contrary, sol-gel Mg-Zr mixed solids exhibit large differences in acidity, depending on its chemical composition: 444, 991 and 766 µmol/g for Mg3Zr-ME, MgZr-ME and MgZr3-ME, respectively. As a rule, surface acidity for ME solids is higher than that obtained for SG series. Finally, it is relevant to note that, in both series, the MgZr solids (with the nominal ratio Mg/Zr = 1) are the most acidic among the whole set of synthesized solids with 991 and 885 µmol/g for MgZr-SG and MgZr-ME, respectively. Moreover, the density of surface acid sites is also the highest for MgZr-SG and Mg-Zr-ME solids with 573 and 449 µmol PY/m^2^, respectively.

As far as the surface basicity is concerned, it was determined by means of carbon dioxide temperature programmed desorption, the results being presented in Table 2 and Supplementary Figures S4 and S5. Regarding the TPD profile, all catalysts except Mg-ME exhibited a similar TPD-CO_2_ profile with a desorption peak centered at 100 °C and a second, truncated, peak associated to the carbon dioxide desorbed during the isothermal period at 200 °C. The Mg-ME solid, however, only presented an intense second peak while the first one is absent for this catalyst. 

As a general rule for the surface basicity per gram of catalyst, ME catalysts present lower basicity than the SG- ones, probably due do the surfactant residues still adsorbed on the catalyst surface. Moreover, it is interesting to point out the low basicity of both pure Mg-SG and Mg-ME solids as compared to the Mg-Zr mixed ones in both series, but especially in the SG-derived catalysts. It has to be assumed that, when calcined at high temperature, MgO is a strongly basic solid that desorbs CO_2_ at temperatures higher than 600 °C. Therefore, since our pure Mg-SG and Mg-ME solids were calcined at 200 °C, they do not present the expected basicity for a pure MgO solid.

If surface basicity per square meter is considered (density of surface basic sites), for both series the obtained results indicate that, as a rule, the higher the amount of magnesium in the solid, the higher the surface basicity (Supplementary Figure S4). In this case, surface basic sites density is higher for Mg-SG and Mg-ME dues to the low surface area of theses solids (41 and 31 m^2^·g^−1^, respectively) indicative of a deficient porous structure as a consequence of the low calcination temperature. 

Moreover, it is also relevant to indicate that Mg3Zr-SG presents a relatively high basicity (7.7 µmol CO_2_/m^2^) when compared with the Mg3Zr-ME solid (2.3 µmol CO_2_/m^2^). For the rest of the catalysts, SG solids exhibit similar surface base properties than their ME counterparts. 

**Figure 6:**
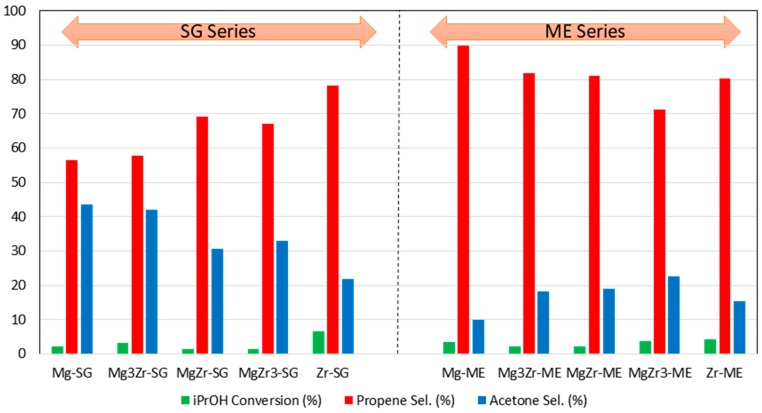
Catalytic activity in the gas-phase propanl-2-ol test reaction for the catalysts prepared in this work. Propan-2-ol conversion and selectivity to propene and acetone obtained at 250 °C.

In addition to the temperature-programmed desorption experiments, surface acid-base properties of all solids were determined through the gas-phase propan-2-ol decomposition test reaction. This reaction allows us to determine acid and basic properties of the catalysts by analyzing the products distribution formed upon propan-2-ol transformation. When the reaction takes place over acid sites, dehydration products (propene and/or diisopropyl ether) are produced, while if basic or redox sites are involved, acetone and/or diacetone alcohol (resulting from acetone self-condensation) are obtained [30,31,32]. Since the catalysts were calcined at 200 °C, initially the reaction was carried out at such temperature observing that there was no propan-2-ol conversion. Therefore, 250 °C was selected as reaction temperature obtaining a propan-2-ol conversion in the 1–7% range. No experiments were carried out at higher temperatures in order to avoid further changes in catalysts structure. Figure 6 shows the result obtained for both series of catalysts.

Despite the low conversion levels obtained, some interesting conclusions could be drawn from these experiments. First of all, for both series of catalysts, pure Zr-SG and Zr-ME catalysts exhibited the highest propan-2-ol conversion with 6.6 and 4.2%, respectively. Pure Mg and mixed Mg-Zr catalysts presented a catalytic conversion in the 1–4% range, irrespective of its composition and synthetic procedure.

The results concerning the selectivity to propene, formed over acid sites, or to acetone, formed over basic ones, seem to be more relevant. Thus, although propene is the major reaction product for all solids with selectivity in the 56–90% range, there are, however, significant differences. In general, in agreement with the TPD-PY results presented above (Table 2 and Supplementary Figure S3), selectivity to propene is higher for the ME- than for the SG series. In this line, the catalysts exhibiting the highest propene selectivity of the whole set of catalysts was Mg-ME (90% selectivity), an unexpected result since this solid was the less acidic (from TPD-PY, Table 2). Probably, the remaining surfactant on the surface of the solids in the ME series accounts for this unexpected result. As for the SG series, propene selectivity results are somehow in good agreement with the TPD-PY data. Thus, pure Zr-SG catalyst presented a 78% selectivity to propene followed by MgZr-SG and MgZr3-SG solids with 69% and 67% selectivity respectively. These were also the most acidic catalysts in the SG series from pyridine temperature-programmed desorption experiments.

On the other hand, as far as the selectivity to basic site-derived products is concerned, SG series gives the best results for the selectivity to acetone (22–44% sel.), being the solids with the highest magnesium content those giving the highest values: Mg-SG and Mg3Zr-SG with 44% and 42% sel., respectively. These results agree with catalyst basicity (TPD-CO_2_, Table 2) where Mg-SG and especially Mg3Zr-SG present the highest basicity among the SG series catalysts. In contrast, for the catalysts corresponding to the ME series, the selectivity to acetone is in the range of 10–23%, even for those solids constituted mostly by Mg species (Mg-ME, 10% sel. to acetone). This fact, together with the unexpectedly high propene selectivity shown by the ME series catalysts, seems to indicate that surfactant molecules still remaining on the catalyst surface after calcination at 200 °C affect its surface chemical properties.

### 2.3. Catalytic Activity 

**Figure 7:**
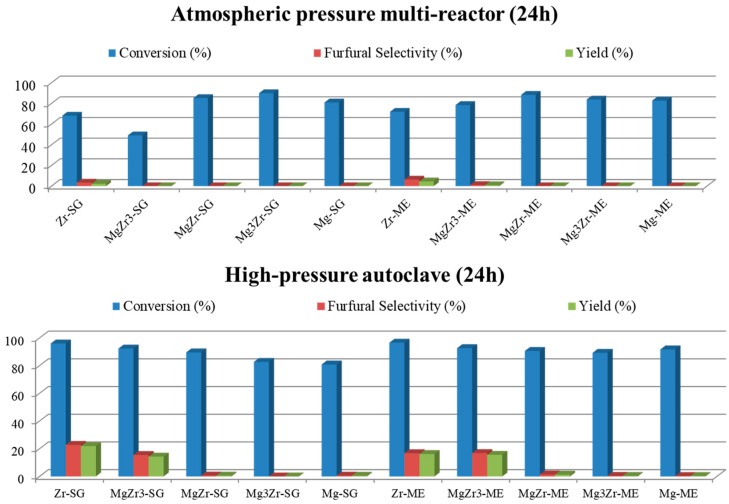
Catalytic activity for both series of catalysts in xylose dehydration in an atmospheric pressure multi-reactor (**upper**) and a high-pressure autoclave (**lower**) in a butan-1-ol/water biphasic media at 150 °C. Xylose conversion and furfural selectivity and yield.

The above described catalysts were tested in the liquid-phase xylose dehydration to furfural in either a high-pressure autoclave or in an atmospheric pressure multi-reactor. The reaction medium in both cases was a biphasic system consisting of an organic/water (1:1 *v*/*v*) mixture, butan-1-ol or toluene being used as the organic solvent. The selected reaction temperature was 150 °C. An initial set of reactions was performed to select the most appropriate reaction system to maximize the furfural yield. Figure 7 shows the catalytic activity obtained for both series of catalysts in a butan-1-ol/water biphasic mixture in a multi-reactor and high-pressure autoclave reactors after 24 h of reaction.

As can be observed in both reaction systems, xylose conversion is higher than 50%, being in most cases close to 90–100%. However, in spite of the above-described high xylose conversion, the selectivity for furfural is quite low (less than 25% at the best). Moreover, as a general trend, xylose conversion as well as furfural selectivity obtained in the high-pressure autoclave are both higher than those obtained in the multi-reactor system. Focusing on the autoclave reactions, Zr-SG and Zr-ME (pure zirconia solids) presented 23% and 17% selectivity to furfural, followed by MgZr3-SG and MgZr3-ME catalysts with 16% selectivity in both cases. As could be expected, the higher the zirconium content, the higher the dehydrating activity for the catalysts. 

**Figure 8:**
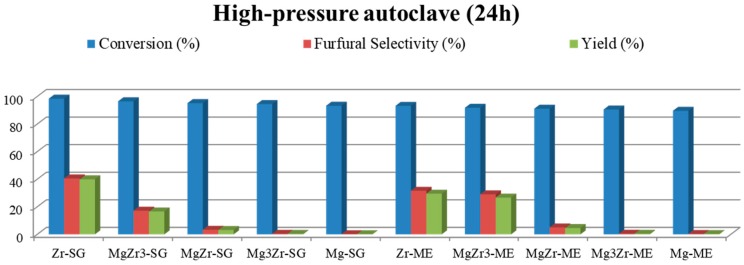
Catalytic activity for both series of catalysts in xylose dehydration in a high-pressure autoclave in a toluene/water biphasic media at 150 °C. Xylose conversion and furfural selectivity and yield.

Based on the above-presented results, additional experiments were carried out with the high-pressure autoclave in order to increase the selectivity to furfural. Therefore, toluene was essayed as organic solvent in the biphasic reaction media, the obtained results being presented in Figure 8. The general trend observed in toluene/water medium is similar to that already reported by using butan-1-ol/water, that is, xylose conversions higher than 90% but, in this case, selectivity values to furfural practically doubled those obtained for the butan-1-ol/water mixture. Thus, Zr-SG presents a 41% selectivity to furfural, followed by Zr-ME (32% sel.), MgZr3-ME (29% sel.) and MgZr3-SG (17% sel.). The rest of the catalysts presented furfural selectivity lower than 5%. 

**Figure 9:**
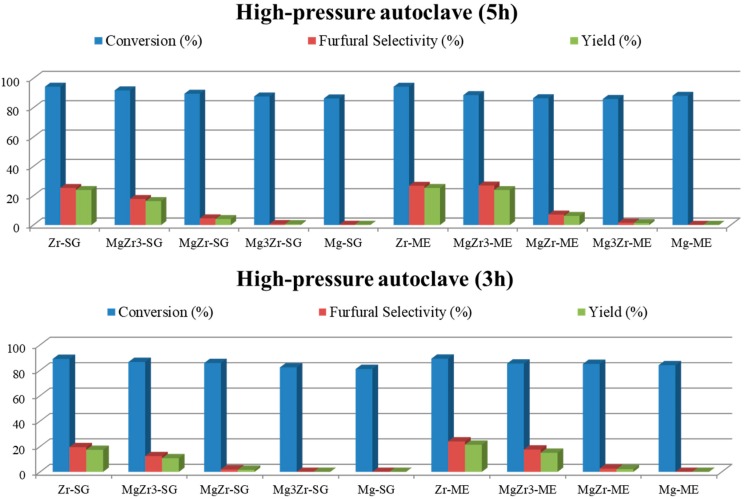
Catalytic activity in xylose dehydration in a high-pressure autoclave in a toluene/water biphasic media at 3 and 5 h of reaction, and 150 °C.

According to Hu et al. [23] ,with aromatic solvents, the formation of furfural from xylose is quick but so is its degradation. Therefore, additional experiments were carried out at shorter reaction times (3 and 5 h) to analyze the influence of reaction time on both xylose conversion and furfural selectivity (Figure 9). Surprisingly, after 3 h of reaction xylose conversion was already higher than 80% indicating that xylose was converted at the very beginning of the reaction process. Moreover, furfural selectivity obtained at shorter reaction time was lower than that reported at 24 h suggesting that xylose was converted to partially dehydrated intermediates during the initial stages of the process. It is interesting to note here that the transformation of xylose into furfural implies the loss of 3 water molecules and, therefore, longer reaction times facilitate furfural formation.

**Figure 10:**
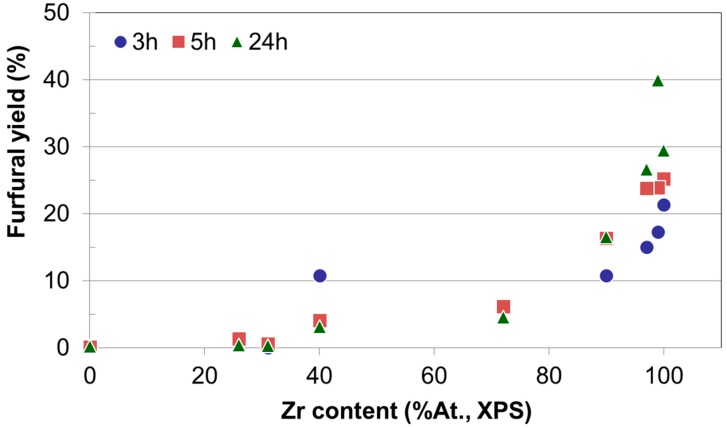
Furfural yield in xylose dehydration as a function of zirconium content (XPS) obtained after 3, 5 and 24 h of reaction at 150 °C in toluene/water reaction medium.

Since the time dependence of xylose conversion and furfural selectivity are opposite, it seems to be relevant to focus the discussion on the yield to furfural for all reaction times and catalytic systems. Moreover, as discussed above, the higher the zirconium content in the catalysts the better the furfural yield achieved. To illustrate this, Figure 10 presents the yield to furfural as a function of zirconium content for all reaction times essayed. It can be observed that irrespective of the reaction time, the yield to furfural depends mainly on the Zr content on the catalyst, being Zr-SG the solid yielding 40% to furfural as the better value after 24 h of reaction. Zr-ME (29% yield), MgZr3-ME (27%) and MgZr3-SG (17%) catalysts showed a reasonably high yield to furfural. On the contrary, solids with high proportion of Mg yielded low amounts of furfural.

The results described above point out that the Zr sites are responsible for the dehydration of xylose to furfural, whereas the Mg related sites are not active at this point. However, in order to extend the scope of the research to cascade (one-pot) reactions, where both acid and base sites are needed, MgZr3 and MgZr catalysts may be interesting solids. Catalysts based on pure MgO, however, appear to be unsuitable for a reaction sequence including a dehydration step, as might reasonably be expected.

Finally, additional experiments were designed to rule out any homogeneous catalytic process due to the possibly lixiviated zirconium species under hydrothermal reaction conditions. Thus, Zr-SG catalyst was added to toluene/water (without xylose) solution and put under reaction condition (6 bar initial N_2_ pressure, 150 °C) for 5 h. Then, the solid was removed by vacuum filtration and the appropriate amount of xylose was added to the filtrated, and put again under reaction conditions for another 5 h of reaction. The xylose conversion obtained in this experiment was similar to that obtained in blank experiments, without any catalyst (less than 7%).

Additional experiments were carried out to verify the recyclability of our catalysts. Several reactions were performed using the MgZr3-SG catalyst; the reactions were stopped after 3 h and the catalyst was recovered by vacuum filtration, washed with distilled water, dried in an oven at 110 °C overnight and calcined at 200 °C before being re-tested in a new reaction. 

**Figure 11:**
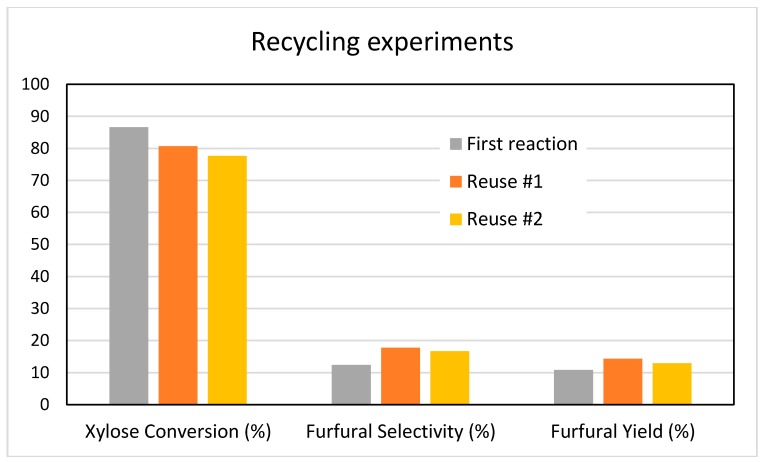
Conversion, selectivity and yield to furfural obtained during the dehydration of xylose on MgZr3-SG catalyst and two subsequent reuses. Reaction conditions: 0.1 g of catalyst, 20 mL of biphasic reaction mixture (water / toluene), 150 °C, 3 h reaction.

Figure 11 shows the results obtained after two reuses of the MgZr3-SG catalyst. With regard to the conversion of xylose (87% conversion in the first reaction), the conversion fell to 81% (a reduction of 7%) while after the second reuse, the conversion obtained was 78%, which represents a reduction of 10% with respect to the initial one. On the contrary, during the reuse tests, the selectivity to furfural increases slightly from the initial 12% to the 17–18% obtained in the reuses and, therefore, the furfural yield increases from 11 to 14%.

In line with the reuse experiments, after 3 h of reaction on MgZr3-SG catalyst, the aqueous phase of the reaction medium was analyzed by ICP-MS to verify the existence of leaching of the component species of the catalyst.

Results indicated the existence of magnesium at a level of 500 ppm while detected amount of zirconium was less than 20 ppb. According to the EDX composition of MgZr3-SG catalyst (Table 1), MgO molar content in the solid was 32.6% and, therefore, 65% of magnesium species were leached to the liquid-phase. However, zirconium species were resistant to leaching under reaction conditions. 

In any case, as reported above, the loss in catalytic activity after the first reuse was only of 7%, while furfural selectivity slightly increases. These results support the conclusion that ZrO_2_ is the catalytically active component of the mixed catalyst.

## 3. Discussion

Two series of catalysts were prepared by sol-gel (SG series) and microemulsion (ME series) synthetic procedure. Each series includes both pure Mg and Zr solids as well as Mg-Zr mixed solids with 25%, 50% and 75% nominal Zr content.

Thermal TGA-DSC experiments carried out to the precursor ME-derived gels of the solids indicate that although the precipitated solid was washed five times with a mixture of chloroform/ethanol, part of the surfactant is retained on the surface of the solid. Moreover, results indicate that the surfactant is retained mainly on the Zr component of the solids, as confirmed by the detection of Br from the surfactant by SEM-EDX.

Due to the low calcination temperature (200 °C), the porous structure of pure MgO-based catalysts has not been adequately developed as evidenced by the low surface area of the Mg-SG and Mg-ME catalysts. This lack of porous structure is reflected in the low surface basicity of these solids. In addition, the catalysts synthesized by ME retain surfactant in their structure which makes them less basic. TEM micrographs indicate that pure Zr-based catalysts are formed by agglomerated of small particles (2–5 nm) while pure Mg-SG and Mg-ME solids are based on larger hexagonal particles of around 200 nm in diameter. In addition, mixed Mg-Zr solids are formed by large MgO particles surrounded by small ZrO_2_ nanoparticles.

In terms of acidity, it is possible that Bromide, coming from the surfactant, is adsorbed on the solids creating Lewis acid sites responsible for relatively higher acidity in the solids of the ME series than in those of the series SG.

The above results clearly indicate that the ME series catalysts retain surfactant adsorbed on their surface after calcination at 200 °C which determines their surface chemical properties. In any case, the catalyst MgZr-SG has the best surface acidic properties, either per gram or per square meter of catalyst. As far as basicity is concerned, the solid with the most promising basic properties is Mg3Zr-SG, although MgZr-SG also shows interesting basic surface properties.

These results are reflected in the propan-2-ol decomposition test reaction since the Mg3Zr-SG catalyst exhibits comparable selectivities to propene and acetone, as a consequence of equilibrium balance between surface acid and basic properties.

The synthesized catalysts were tested for the xylose dehydration reaction in two different reaction systems (high-pressure autoclave and atmospheric-pressure multi-reactor) and using different reaction mixtures (solvents). For both reaction systems and using butan-1-ol/water as solvent, the obtained xylose conversions after 24 h of reaction were high, although the furfural selectivity obtained was quite low (less than 25% sel.). In any case, the yields to furfural were higher in the high-pressure autoclave, so the multi-reactor at atmospheric pressure was ruled out for further testing. Similar yields were reported in the literature for monosaccharides dehydration on acid-base heterogeneous catalysts such as CoAl-hydrotalcite (22% yield to hydroxymethylfurfural) [33].

As regards the solvent effect, the change from butan-1-ol to toluene as the organic solvent in the biphasic reaction mixture led to better yields to furfural after 24 h of reaction. According to the literature, compared with water, alcohols can promote the formation of furfural and slow down its degradation at prolonged reaction times. Moreover, when using aromatics as organic solvent, the formation of furfural is quick but so is the degradation of furfural due to the aprotic properties of these solvents [23]. This behavior is, however, contrary to that observed in our case since with toluene better yield to furfural is obtained as compared to butan-1-ol (Zr-SG, toluene/water, 24 h, 40% yield vs. Zr-SG, butan-2-ol/water, 24 h, 22% yield). This could be due to the fact that toluene is a good organic solvent that can extract furfural as it is formed, thus avoiding the formation of humins which takes place mainly in the aqueous phase [23].

Additional tests conducted with toluene/water at shorter reaction times (3 and 5 h) still led to high xylose conversions but associated with even lower furfural selectivities. This behavior could be associated with a rapid dehydration of xylose to partially dehydrated intermediates which, along the reaction course, slowly culminate in the formation of furfural.

When we study the influence of the catalyst on the yield to furfural it is observed that the higher the zirconium content, the higher the dehydrating activity for the catalysts. That is, irrespective of the reaction time, the yield to furfural depends on the Zr content on the catalyst, while, on the contrary, solids with high proportion of Mg yielded low amounts of furfural (0% yield for Mg-SG and Mg-ME catalysts).

Finally, the reuse experiments of the catalysts, together with the analysis of leached species, indicated that while ZrO_2_ component is practically not leached, a large part of the MgO component of the solids is leached to the reaction medium under the hydrothermal reaction conditions. However, this only implies a slight reduction in the conversion of xylose because, as mentioned, it is the ZrO_2_ component that is responsible for the catalytic activity of the MgO-ZrO_2_ mixed oxides.

All in all, from the point of view of the xylose dehydration to furfural, the catalysts constituted by pure ZrO_2_ (especially Zr-SG) are the most suitable to carry out the process under study.

However, if a second process that occurs with the participation of surface basic sites is to be coupled to it in the so-called one-pot or cascade reactions, the MgZr mixed solids could be suitable for the overall process. Thus, the MgZr-SG and MgZr3-SG catalysts present a balance of acid and base centers that could be adequate for this type of processes. Leaching of the MgO component, however, is an issue that has to be solved in order to carry out the process in an effective way. 

Finally, although some catalysts of the series ME present results that could be interesting, the problems associated to the surfactant that remains adsorbed on them seem to advise its use in more complex processes.

## 4. Materials and Methods 

### 4.1. Materials

Chloroform anhydrous ≥99% (ref. 288306), 2,2,4-trimethylpentane anhydrous 99.8% (ref. 360066), hexadecyltrimethylammonium bromide ≥98% (CTAB) (ref. H5882), butan-1-ol ≥99% (ref. B7906), ethanol (ref. 652261), zirconium (IV) oxynitrate hydrate (ref. 346462), magnesium nitrate hexahydrate (ref. 203696), d-(+)-xylose ≥99% (ref. X1500) and toluene (ref. 244511) were purchased from Sigma-Aldrich (Merck KGaA, Darmstadt, Germany). Ammonium hydroxide solution 5.0 N (ref. 318612) and hydrochloric acid solution 1.0 M (ref. 318949) were obtained from Honeywell Fluka (Honeywell, Bucharest, Romania). MilliQ water was used for preparation of the aqueous solutions.

### 4.2. Catalysts Synthesis

Pure MgO and ZrO_2_ as well as several mixed MgO-ZrO_2_ systems (in an atomic Mg:Zr nominal ratio of 75:25, 50:50 and 25:75) were synthesized through both microemulsion (ME series) and sol-gel (SG series) methods. In both synthetic methods, magnesium and zirconyl nitrates were used as the precursors and aqueous NH_4_OH (pH 10) as precipitating agent.

#### 4.2.1. Sol-Gel Method

MgO was prepared from a 0.4 M aqueous solution of magnesium nitrate hexahydrate which was introduced at controlled rate (0.56 mL/min) into a reactor containing 0.4 L of ammonium hydroxide solution at pH 10. The process was carried out under magnetic stirring (700 rpm) and maintaining the initial pH level of 10 with help of a Syrris Atlas pump (Syrris Ltd., Hertfordshire, UK) that introduced NH_4_OH 5 N or HCl 0.2 M as needed. The gel was aged for 24 h and the precipitate was then vacuum filtered and washed with water to obtain a white solid which was dried overnight at 120 °C. The dried solid was calcined at 200 °C for 6 h under air flow. Finally, the solid was ground and sieved (0.149 µm light sieve). Similar procedure was followed for the rest of catalysts in the SG series just adjusting the appropriate amounts of magnesium and zirconium precursors to obtain solids with Mg:Zr atomic ratio of 100:0 (Mg-SG), 75:25 (Mg3Zr-SG), 50:50 (MgZr-SG), 25:75 (MgZr3-SG) and 0:100 (Zr-SG). 

#### 4.2.2. Microemulsion Method 

As for the solids prepared through microemulsion technique (ME series), two different microemulsions were prepared to include Mg and Zr precursors (ME1) and the precipitating agent, NH_4_OH (ME2). Under optimized conditions, the composition of microemulsions was: 2,2,4-trimethylpentane anhydrous (oil, 53 wt %), hexadecyltrimethylammonium bromide (surfactant, 15 wt %), 1-butanol (co-surfactant, 12 wt %) and 0.4 M aqueous solution of Mg and Zr precursors (ME1) or NH_4_OH aqueous solutions of pH = 10 (ME2) (20 wt %). 

As a standard procedure ME1 was slowly added to ME2 under constant stirring (700 rpm) and the resulting mixture aged overnight under stirring. The white precipitate was then filtered and washed five times with 80 mL of a chloroform/ethanol mixture (1:1 *v*/*v*) to remove surfactant and oil remaining on the solid surface. The solid was dried, calcined and sieved in a similar way to the SG series. Catalysts in the ME series were prepared just adjusting the appropriate amounts of magnesium and zirconium precursors to obtain solids with Mg:Zr atomic ratio of 100:0 (Mg-ME), 75:25 (Mg3Zr-ME), 50:50 (MgZr-ME), 25:75 (MgZr3-ME) and 0:100 (Zr-ME).

### 4.3. Catalysts Characterization

Both series of catalysts were thoroughly characterized from textural, structural and chemical point of view. 

Thermogravimetric analyses (TGA) were performed on a Setaram SetSys 12 instrument (Caluire, France). An amount of 20 mg of sample was placed in an alumina crucible and heated at temperatures ranging from 30 to 600 °C (heating rate of 10 °C·min^−1^) under synthetic air stream (50 mL·min^−1^) in order to measure weight loss, heat flow and derivative weight loss.

Surface areas of solids were obtained from nitrogen adsorption-desorption isotherms obtained at liquid nitrogen temperature on a Micromeritics ASAP-2010 instrument, following the Brunnauer-Emmett-Teller (BET) method. All samples were degassed to 0.1 Pa at 120 °C before measurement.

Scanning electron microscopy (SEM-EDX) measurements were obtained on a JEOL JSM-6300 scanning electron microscope (Jeol, Tokyo, Japan) equipped with an energy-dispersive X-ray (EDX) detector (Oxford Instruments, Abingdon, United Kingdom), available at the Central Service for Research Support (SCAI) of the University of Córdoba. It was operated at an acceleration voltage of 20 keV with a resolution of 65 eV. Transmission electron microscopy (TEM) images were obtained at the Central Service for Research Support (SCAI) of the University of Córdoba using a JEOL JEM 1400 microscope (Jeol, Tokyo, Japan). All samples were mounted on 3 mm holey carbon copper grids.

X-ray photoelectron spectroscopy (XPS) data were recorded at the Central Service for Research Support (SCAI) of the University of Córdoba on 4 mm × 4 mm pellets, 0.5 mm thick that were obtained by gently pressing the powered materials. Samples were outgassed to a pressure below about 2 × 10^−8^ Torr at 150 °C in the instrument pre-chamber to remove chemisorbed volatile species. The main chamber of the Leibold-Heraeus LHS10 spectrometer used (Leibold, Cologne, Germany), capable of operating down to less than 2 × 10^−9^ Torr, was equipped with a EA-200MCD hemispherical electron analyzer with a dual X-ray source using Al Kα (hυ = 1486.6 eV) at 120 W and 30 mA. C (1s) was used as energy reference (284.6 eV).

Surface acidity of the catalysts was determined by thermal programmed desorption of pre-absorbed pyridine (TPD-PY) with TCD detection. An amount of 20 mg of sample was introduced in a 10 mm ID reactor that was placed inside an oven. Solids were cleaned under He flow (75 mL·min^−1^) by heating to 200 °C at a rate of 10 °C min^−1^ and then cooled down to 50 °C. At that temperature, the solids were exposed for 30 min to a pyridine saturated Helium flow. After saturation, physisorbed pyridine was removed by flowing a pure He stream for 60 min (75 mL·min^−1^). Then, the temperature-programmed desorption of chemisorbed pyridine was carried out by ramping the temperature from 50 to 200 °C (heating rate 10 °C·min^−1^) and holding the final temperature for 30 min. Desorbed pyridine was quantified against a calibration graph constructed from variable volumes of pyridine injected.

Surface basicity of the catalysts was determined on a Micromeritics Autochem II instrument (Micromeritics, Norcross, GA, USA) by thermal programmed desorption of pre-absorbed CO_2_ (TPD-CO_2_) with TCD detection. An amount of 100 mg of each catalyst was loaded into a reactor 10 mm ID and placed in a furnace. Solids were cleaned with an Ar stream (20 mL·min^−1^) by heating to 200 °C at a rate of 10 °C·min^−1^ for 60 min and then cooled down to 40 °C. At that temperature, the catalysts were saturated with the probe molecule, using 5%CO_2_/Ar flow at 20 mL·min^−1^ for 60 min. After saturation, physisorbed CO_2_ was removed by flowing Ar stream for 30 min (20 mL·min^−1^). Then, the temperature-programmed desorption of chemisorbed CO_2_ was carried out by ramping the temperature from 40 to 200 °C (heating rate 5 °C·min^−1^) and holding the final temperature for 60 min. The amount of CO_2_ adsorbed was determined from a calibration graph constructed from the injection of variable volumes of 5%CO_2_/Ar.

Propan-2-ol transformation test reaction was also used to confirm the surface chemical properties of the prepared catalysts. This reaction, widely described as a model process, can provide valuable information on surface acid-base properties of the catalyst as a function of its products distribution: surface acid sites yield propene or diisopropyl ether while basic or redox properties lead to acetone [30,31,32,34,35]. 

The gas-phase propan-2-ol transformation was carried out in a stainless-steel reactor (1/8 inch OD) that was loaded with 100 mg of catalyst and 1 g of inert SiO_2_. Prior to the reaction, the catalyst was cleaned with a N_2_ flow (20 mL·min^−1^) while increasing the temperature until 200 °C (rate, 2 °C·min^−1^). The reaction was started by introducing propan-2-ol by passing a nitrogen flow of 10 mL·min^−1^ through a saturator filled with propan-2-ol at room temperature. Analyses were carried out on-line by connecting the effluent to a gas chromatograph (HP 5890 series II) equipped with a capillary column Supelcowax-10 (60 m long, 0.25 mm ID, 0.25 μm film thickness).

### 4.4. Catalytic Tests

The solids were tested for dehydration of xylose to furfural using a biphasic reaction medium on two different reaction systems.

#### 4.4.1. Multi-Reactor under Conventional Thermal Heating

A Carousel 12 Reaction StationTM multi-reactor (Radley Discovery Technologies, Essex, UK) furnished with twelve reaction glass tubes with a maximum reaction volume of 20 mL. The reaction mixture consisted of 75 mg xylose, 6 mL milliQ water, 6 mL organic solvent (1-butanol or toluene) and 30 mg catalyst. The reaction mixture was purged with N_2_ before reaction to work under controlled atmosphere. Temperature and stirring rate were adjusted at 150 °C and 750 rpm, respectively, and the reaction time extended to 24 h. The upper part of the reaction tubes was always refrigerated at 5 °C thus preventing loss of the reaction mixture by evaporation.

#### 4.4.2. High-Pressure Autoclave

A Berghof HR-100 stainless steel high-pressure autoclave (Berghof Products and Instruments GmbH, Eningen, Germany) equipped with a 75 mL PTFE insert vessel was also used in xylose dehydration to furfural. Under standard conditions, 250 mg xylose, 10 mL milliQ water, 10 mL organic solvent (1-butanol or toluene) and 100 mg catalyst were introduced in the vessel (i.e., same substrate/catalyst ratio as in multi-reactor). Reactor was purged with nitrogen and pressurized to 5 bar of N_2_. The reaction temperature was set to 150 °C and the reaction started by switching on the stirring at 750 rpm. Reaction was stopped by introducing the vessel in an ice bath at selected times (3, 5 and 24 h). 

#### 4.4.3. Product Analysis

Once finished the reactions, the aqueous and organic phases were separated by centrifugation and filtered through a nylon syringe filter (0.22 µm). The aqueous phase was analysed by high-performance liquid chromatography (Water 2695 HPLC) with refraction index detection (IR detector) on a Fortis amino 5 µm (250 × 4.6 mm) column. 5 mM of H_2_SO_4_ was employed as the eluent with a 0.7 µL/min flow rate at 50 °C. The volume of injection was 5 µL. The organic phase was analysed by gas chromatography (Agilent 7890, Santa Clara, CA, USA) with flame ionization detector (GC-FID) using a Supelco Nukol™ capillary column. No xylose was detected in the organic phase and furfural detected in the aqueous phase was negligible. Quantification of xylose and furfural was performed using the appropriate calibration curves.

Blank experiments (without catalyst) were carried for both reaction systems being the xylose conversion obtained less than 7% at the best.

Xylose conversion and furfural selectivity and yield were defined by Equations (1)–(3):

(1) $$\mathrm{Xylose}\;\mathrm{conversion}\;\left(\%\right)\;=\;\frac{initial\;xylose\;cncentration-final\;xylose\;cncentration}{initial\;xylose\;cncentration}\times 100$$

(2) $$\mathrm{Furfural}\;\mathrm{selectivity}\;\left(\%\right)=\frac{furfural\;cncentration}{initial\;xylose\;cncentration-final\;xylose\;cncentration}\times 100$$

(3) $$\mathrm{Furfural}\;\mathrm{yield}\;\left(\%\right)=\frac{furfural\;cncentration}{initial\;xylose\;cncentration}\times 100$$

## 5. Conclusions

Two series of catalysts were prepared (by sol-gel and microemulsion synthetic procedures), characterized and applied to the liquid-phase xylose dehydration to furfural. Catalysts prepared by microemulsion retained part of the surfactant used in the synthetic procedure, mainly associated to the Zr part of the solid. 

As for xylose dehydration, the use of toluene as organic solvent in the biphasic reaction mixture (toluene/water) leads to better performance to furfural than the use of alcohols (1-butanol) which is associated with the ability of toluene to extract the furfural formed, thus avoiding the formation of humins that take place mainly in the aqueous phase. 

The yield to furfural increases with the Zr content of the catalyst and, therefore, the catalysts constituted by pure ZrO_2_ (especially Zr-SG) are the most suitable to carry out the process (98% conversion, 40% yield, 24 h). The results described above point out that the Zr sites are responsible for the dehydration of xylose to furfural, whereas the Mg related sites are not active at this point. 

The reuse experiments of the catalysts, together with the analysis of leached species, indicated that while ZrO_2_ component is practically not leached, a large part of the MgO component of the solids is leached to the reaction medium under the hydrothermal reaction conditions. However, this only implies a slight reduction in the conversion of xylose because, as mentioned, it is the ZrO_2_ component that is responsible for the catalytic activity of the MgO-ZrO_2_ mixed oxides.

## Figures and Tables

**Table 1:** Nominal, EDX and XPS chemical composition (%mol) and textural properties of the catalytic systems synthesized in this work.

	Chemical Composition (Nominal)		Chemical Composition (EDX)		Chemical Composition (XPS)		Surface Area
**Catalyst**	**Mg**	**Zr**	**Mg**	**Zr**	**Mg**	**Zr**	**BET (m^2^/g)**
Mg-SG	100	0.0	100	0.0	100	0.0	41
Mg3Zr-SG	75	25	94.2	5.8	69.4	30.6	119
MgZr-SG	50	50	67.7	32.3	60.3	39.7	173
MgZr3-SG	25	75	32.6	67.4	10.4	89.6	169
Zr-SG	0.0	100	0.0	100	0.0	100	239
Mg-ME	100	0.0	100	0.0	100	0.0	31
Mg3Zr-ME	75	25	90.5	9.5	74.2	25.8	196
MgZr-ME	50	50	70.6	29.4	27.4	72.6	197
MgZr3-ME	25	75	22.2	77.8	3.5	96.5	192
Zr-ME	0.0	100	0.0	100	0.0	100	222

**Table 2:** Catalysts Surface acid-base properties obtained from temperature programmed desorption of pyridine (TPD-PY) and carbon dioxide (TPD-CO_2_) experiments.

Catalyst	Catalyst Acidity (TPD-PY)		Catalysts Basicity (TPD-CO_2_)	
	µmol Py/g	µmol Py/m^2^	µmol CO_2_/g	µmol CO_2_/m^2^
Mg-SG	176	4.36	457	11.29
Mg3Zr-SG	444	3.73	918	7.71
MgZr-SG	991	5.73	526	3.04
MgZr3-SG	766	4.53	517	3.06
Zr-SG	748	3.13	441	1.84
Mg-ME	115	3.69	354	11.42
Mg3Zr-ME	856	4.37	448	2.28
MgZr-ME	885	4.49	292	1.48
MgZr3-ME	788	4.11	368	1.91
Zr-ME	871	3.92	199	0.90

## Supplementary Materials

The following are available online: Supplementary Figure S1: SEM-EDX measurements for the MgZr-ME catalysts, including EDX profiles for the whole image area. Inserts correspond to the chemical composition associated to EDX profile. Red arrow marks Br signal, Supplementary Figure S2: Nitrogen adsorption-desorption isotherms corresponding to the solids synthesized in both SG and ME series, Supplementary Figure S3: Catalysts acidity determined from temperature programmed desorption of pre-adsorbed pyridine (µmol PY/g CAT and µmol PY/m^2^ CAT), Supplementary Figure S4: Catalysts basicity determined from temperature programmed desorption of pre-adsorbed carbon dioxide (µmol CO_2_/g CAT and µmol CO_2_/m^2^ CAT), Supplementary Figure S5: Temperature-programmed desorption profiles of pre-adsorbed carbon dioxide obtained for the catalysts synthesized in this work.
