# Supplementary material for: Microemulsion and Sol-Gel Synthesized ZrO2-MgO Catalysts for the Liquid-Phase Dehydration of Xylose to Furfural
Source: Molecules. 2017 Dec 18;22(12):2257. doi: 10.3390/molecules22122257 (PMC6150008; doi:10.3390/molecules22122257)
Supplement: Supplementary file 1 [file molecules-22-02257-s001.pdf]

molecules

MDPI

Article

Microemulsion and sol-gel synthesized ZrO_2-MgO catalysts for the liquid-phase dehydration of xylose to furfural

Almudena Parejas[1], Vicente Montes[1], Jesús Hidalgo[1], Elena Sánchez[1], Alberto Marinas[1] and Francisco J. Urbano [1,*]

[1] Departamento de Química Orgánica, Instituto Universitario de Investigación en Química Fina y Nanoquímica IUIQFN, Universidad de Córdoba, Campus de Rabanales, Edificio Marie Curie, E-14014 Córdoba, España

* Correspondence: fj.urbano@uco.es; Tel.: +34-957-218-638

Academic Editor: name

Received: date; Accepted: date; Published: date

Supplementary Materials: The following are available online at www.mdpi.com/link,

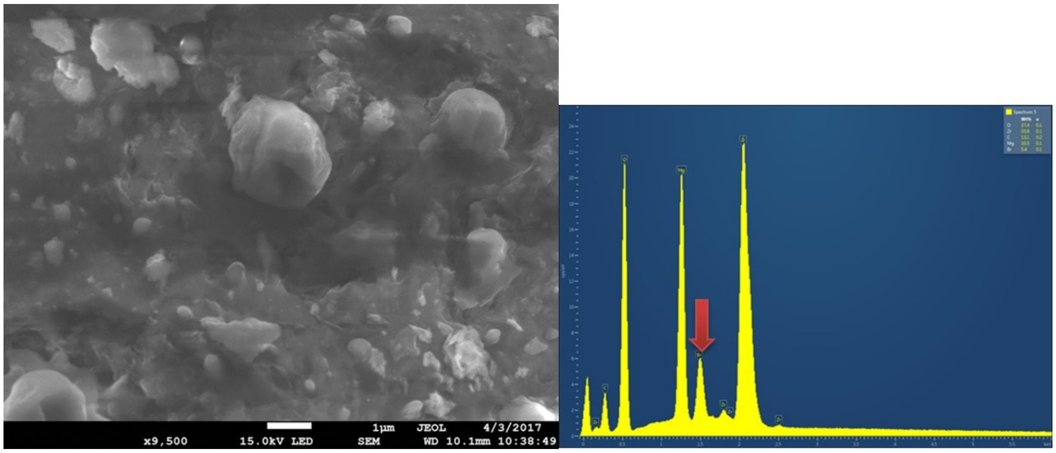

Supplementary Figure S1: SEM-EDX measurements for the MgZr-ME catalysts, including EDX profiles for the whole image area. Red arrow marks Br signal (2.1% atomic).

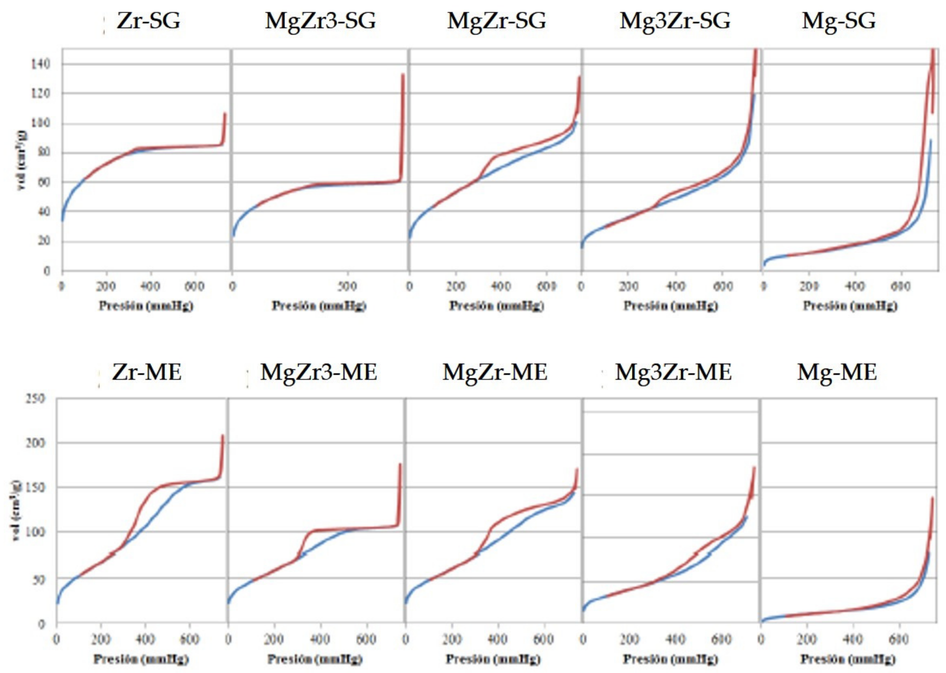

Supplementary Figure S2: Nitrogen adsorption-desorption isotherms corresponding to the solids of both SG and ME series.

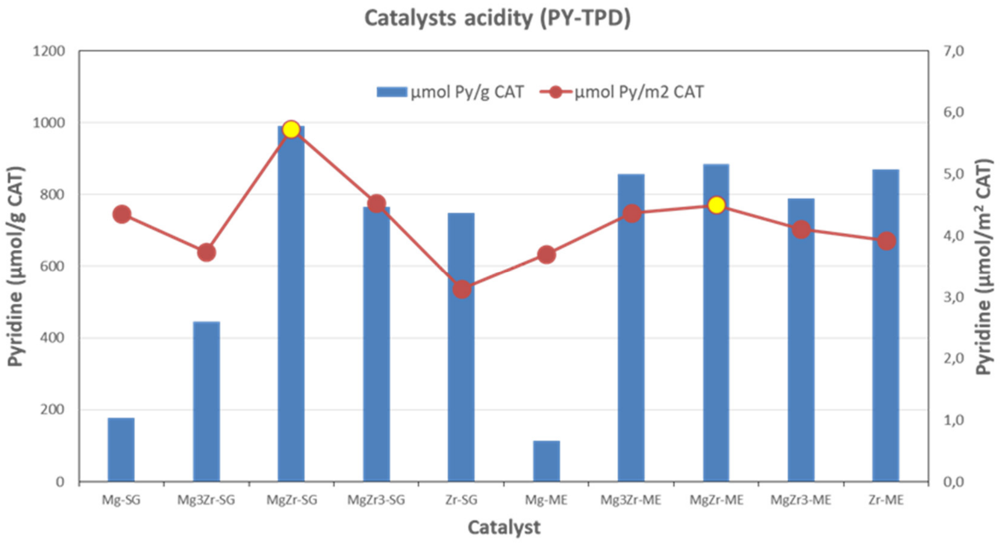

Supplementary Figure S3: Catalysts acidity determined from temperature programmed desorption of pre-adsorbed pyridine (µmol PY/g CAT and µmol PY/m² CAT).

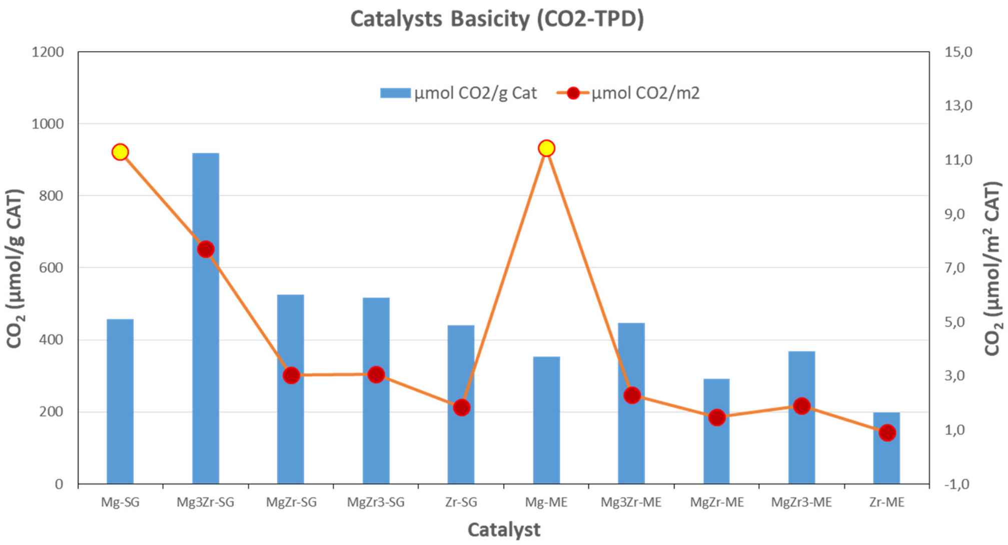

Supplementary Figure S4: Catalysts basicity determined from temperature programmed desorption of pre-adsorbed carbon dioxide (µmol CO_2/g and µmol CO_2/m^2).

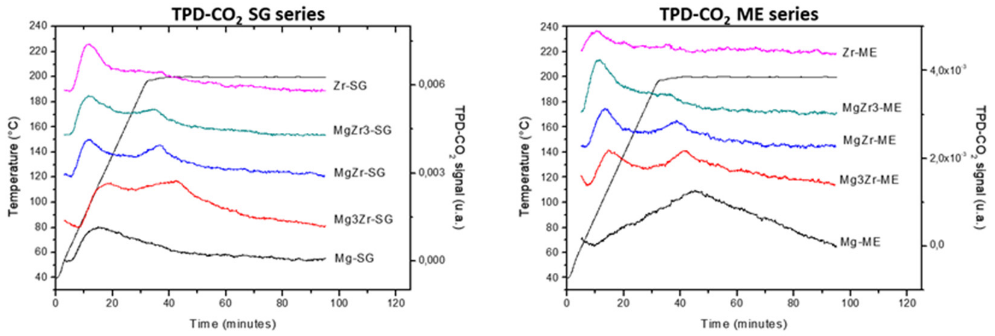

Supplementary Figure S5: Temperature-programmed desorption profiles of pre-adsorbed carbon dioxide obtained for the catalysts synthesized in this work.
